# Technical efficiency of the urban gas industry in china

**DOI:** 10.1007/s11356-023-29524-3

**Published:** 2023-09-13

**Authors:** Fan Li, Xiaokun Wu, Michelle Andrea Phillips

**Affiliations:** 1 China Center for Special Economic Zone Research, Shenzhen University, Nanhai Ave 3688, Shenzhen, 518060 Guangdong China; 2 Economics Department, University of Florida, Gainesville, USA; 3 W. P. Carey School of Business, Arizona State University, Tempe, USA

**Keywords:** Urban gas, Natural gas, Petroleum, Stochastic Frontier Analysis (SFA), Technical efficiency, Cobb Douglas production function, L51, L95

## Abstract

China’s Paris Agreement Pledge and recent introduction of an Emissions Trading Scheme have created a need for information on where it makes the most economic sense to use different sources of energy. With lower carbon dioxide emissions, natural gas and liquefied petroleum gas provide cleaner sources of energy relative to coal. Although it is practically crucial to investigate the use of these two sources, empirical studies are limited due to lack of data. To fill the research gap, this paper studies the efficiency of natural gas and liquefied petroleum gas sector in China by using data from 24 major province-level divisions over the years 2006–2015. Efficiency is defined as the utility’s ability to produce the highest output given fixed inputs. We find that: (1) GDP per capita and high customer density are associated with higher levels of efficiency and (2) utilities that serve higher proportions of households (as opposed to industry and firms) are associated with lower levels of efficiency. Policy makers can use this information to address China’s energy needs due to rapid urbanization while also pursuing Paris Agreement goals.

## Introduction

According to China’s Paris Agreement pledge, the country will see carbon emissions peak by 2030 and achieve net zero emissions before 2060 (Carbon Brief 2021). China expects to achieve the “dual carbon” through the use of an Emissions Trading Scheme (ETS). Emissions Trading Schemes are a preferred policy of economists because they allow for emissions reductions in a way that is cost-effective. For example, if a country has a power plant that can reduce pollution for $12 per pound and a power plant that can reduce it for $5.00 per pound, it makes more financial sense to reduce pollution for $5.00 per pound. With an ETS, market forces make it so the firms with the lowest cost of abatement per pound reduce emissions the most. China’s ETS, launched in 2021, currently covers the power sector and is expected to expand to other sectors in the future (International Carbon Action Partnership 2021).

To meet its “dual carbon” objectives, China has made significant progress in transitioning from coal dependency to low-carbon energy sources. While renewable energy plays a crucial role in reducing carbon emissions and air pollution, it has limitations in terms of storage capacity and geographic availability. Consequently, the composition of the energy mix, i.e., the balance between different energy sources in the supply, has become increasingly important during China's energy transition. Notably, liquefied petroleum gas (LPG) and natural gas offer cleaner energy alternatives to coal, with lower carbon dioxide emissions per million BTUs (EIA 2018). As a result, they have become essential components in the production of low-carbon energy. Moreover, the consumption of natural gas in China continues to rise due to the country’s rapid urbanization and the existing energy infrastructure in industrial and residential sectors. According to a BP report (BP 2018), natural gas has emerged as the largest contributor to China's primary energy growth, accounting for 3.0% of the increase. As of 2020, natural gas consumption in China represented 8.4% of its overall primary energy consumption (Shi 2022). However, it is crucial to acknowledge that natural gas extraction is associated with methane leaks, which have a more potent impact on climate change relative to carbon dioxide, albeit being relatively short-lived. The Intergovernmental Panel on Climate Change (IPCC 2014) highlights that methane generates 86 times more radiative forcing than CO2 within the first 20 years. Consequently, it is imperative to quantify the magnitude of methane emissions throughout the natural gas supply chain and ensure the efficient utilization of natural gas to minimize extraction activities.

With China's commitment under the Paris Agreement, its substantial growth in gas consumption, and the need to regulate natural gas extraction, understanding the regions where gas usage is efficient becomes critical. Efficiency in this context is defined as the firm’s ability to produce the largest amount of output given its input endowment.

Research studying the efficiency of the Chinese urban gas distribution sector is scarce. Studies of the sector have previously focused on price reform (Lin and Wang 2012; Paltsev and Zhang 2015) and policy reform (Dong et al. 2017). The purpose of this paper is to examine the effect of the regulatory environment on the performance of the urban gas sector in China and to characterize factors that encourage efficiency. If China is to reduce its use of coal and fossil fuels in general, while keeping some gas use, it is important to understand under what circumstances the use of gas is efficient. Understanding gas efficiency can help the country decide where gas should be used, and plan for increased energy growth in urban areas with the Paris Agreement goals in mind.

The rest of this paper is organized as follows. “Background: Chinese urban gas sector” section describes the background of the Chinese urban gas sector with a focus on its regulatory environment. “Efficiency studies of oil and natural gas firms” section reviews the literature on the efficiency of gas distribution. “Model” section explains the methodology adopted. In “Empirical analysis” section, we present the efficiency estimation result. Finally, “Concluding observations and policy implications” section concludes and draws policy implications for efficiency improvement.

## Background: Chinese urban gas sector

LPG and natural gas are two main products of the Chinese urban gas sector, accounting for 41.95% and 56.53% respectively in 2016. China has a large amount of natural gas deposits and has been steadily increasing its use of natural gas. Natural gas output in China increased by 12.1% annually between 2000–2014, compared to a 2.6% average annual growth rate worldwide, with natural gas consumption averaging an annual growth rate of 15.3% (Dong et al. 2017). Natural gas in China is mainly distributed in the Midwest and tends to be deeply buried; its exploration and distribution is costly and requires advanced technologies. Compared to natural gas, oil in China is more widely distributed.

According to Dong et al. (2017), the Chinese gas sector has gone through several major stages of reform. These started with a highly centralized planned economic system (1949–1981) and culminated with a separation of government administration from the management of enterprises (1998-present). The National Energy Administration was established in 2008 to handle management functions. There are several other government organizations involved in the administration and regulation of oil and natural gas resources, including the National Development and Reform Commission, the Ministry of Land and Resources, and the Ministry of Environmental Protection. Regulation of oil and natural gas is, thus, highly fragmented, since it is divided up into several different government departments whose role is much larger than the regulation of the gas sector (Dong et al. 2017). This approach sharply differs from regulatory patterns seen in the United States of America and Britain, where regulation of oil and natural gas is carried out by independent regulatory agencies whose sole focus is typically the energy sector or a specific set of utilities. In other words, the Chinese gas sector lacks independence from the government because the regulatory agencies are part of other government agencies.

The natural gas downstream market has gone through two reforms. During the 1978–2002 period, the market consisted of state-owned monopolies in charge of pricing, subsidies, network building and distribution, and marketing. In 2002, the government started relaxing restrictions and allowing for private and foreign capital for facility construction and operation, mostly via franchises and joint ventures (Dong et al. 2017).

Most Chinese gas distributors are owned and managed by local governments (Higashi 2009). In this study, we examine the province-level performance of gas distribution companies. China has jurisdiction over 22 provinces, 5 autonomous regions, 4 direct-controlled municipalities (Beijing, Chongqing, Shanghai and Tianjin) and 2 special administrative regions (Hong Kong and Macau).^1^ The province-level divisions^2^ are collectively known as mainland China which usually excludes the 2 special administrative regions. Due to data availability, our dataset includes 3 direct-controlled municipalities (Chongqing is excluded), 2 autonomous regions (Xizang, Ningxia, Neimenggu are excluded) and 19 provinces (Fujian, Liaoning and Qinghai are excluded). The main province-level divisions of mainland China are included in our dataset, providing a sample of the majority of the Chinese urban gas sector.

## Efficiency studies of oil and natural gas firms

Efficiency analysis of utilities has been studied using a variety of techniques. Studies typically use Data Envelopment Analysis (e.g., Marques et al. 2013; Ohene-Asare et al. 2017), a non-parametric technique or Stochastic Frontier Analysis, a parametric technique. The majority of Stochastic Frontier Analysis studies of the infrastructure sector examine the water (Li and Phillips 2016; Phillips 2013), electricity (Chen et al. 2015; Li et al. 2019), and transportation (Filippini et al. 2015) sectors. There are advantages and disadvantages to both techniques. Stochastic Frontier Analysis allows for statistical inference and allows for noise but requires the imposition of a functional form. Data Envelopment Analysis does not require the researcher to impose a distributional functional form but does not allow for statistical inference.

The efficiency of gas distribution firms has been widely analysed in different countries, such as Turkey (Yardımcı and Karan 2015; Ertürk and Türüt-Aşık 2011), Argentina (Rossi 2001), Italy (Erbetta and Rappuoli 2008; Goncharuk and lo Storto 2017), Ukraine (Goncharuk and lo Storto 2017), America (Hollas et al. 2002), Brazil (Tovar et al. 2015), Netherlands, the UK, and Slovenia (Zorić et al. 2009). Goncharuk and lo Storto (2017), for instance, adopt a 2-stage DEA procedure to evaluate the efficiency of gas companies in Italy and Ukraine under the background of gas reform. Their results suggest that technical and scale efficiency are low in both countries. Ertürk and Türüt-Aşık (2011) study the efficiency of Turkish natural gas distribution companies using Data Envelopment Analysis and find that public firms, non-tender firms, and large firms which operate in more developed areas are more efficient. Rossi (2001) studies the efficiency of the natural gas distribution sector in Argentina in the post-privatization period using a Stochastic Frontier Analysis model and finds that the sector improves its overall performance during this period. Erbetta and Rappuoli (2008) adopt Data Envelopment Analysis to study technical and scale efficiency of Italian gas distribution industry. They find that the technical efficiency level is low over the period 1994–1999 and conclude that intensification of the merging process involving distributors of small scale can improve productivity.

To explore factors that affect efficiency, many studies have investigated the impact of regulatory environment, price system and operational management on the performance of gas firms. Hollas et al. (2002) examine technical efficiency, economies of scale, and efficiency changes for gas distribution utilities in America and find that federal legislative and Federal Energy Regulatory Commission policies to introduce competition lead to a reduction in scale but have no impact on the economic efficiency of the utilities. Hammond et al. (2002) investigate the relative techinical efficiency for inter-war British gas industry and find that utilities operating under the basic price system are of more efficiency than those under the maximum price and the sliding scale regulation system. Tovar et al. (2015) investigate the gas distribution firms’ technical efficiency in Brazil and identify the factors affecting efficiency. They show that large consumer density, the private ownership (vs. public ownership) and price cap regulation (vs. cost of service regulation) are associated with higher level of efficiency.

There are a few studies seeking to investigate the efficiency of the Chinese gas sector as a whole, however, little attention has been paid to the gas distribution sector. Eller et al. (2011) study the operational efficiency of national oil companies from several countries using both Stochastic Frontier Analysis and Data Envelopment Analysis and find that national oil companies are less efficient than international oil companies. They also find that a large amount of inefficiency can be explained by structural and institutional features of firms. Their sample includes three Chinese oil firms. Hu (2014) evaluate energy efficiency for 150 plants from China’s energy sector for the period 2000–2005, but he does not explore the factors that are related to inefficiency.

Filling the research gap, in this paper, we examine the efficiency of production functions in the gas distribution sector of China for the more recent period 2006–2015. Moreover, we investigate the effect of the regulatory environment on the performance of the urban gas firms and aim to address factors that relate to technical efficiency in the Chinese gas sector.

## Model

We use a Stochastic Frontier Analysis (SFA) model to examine the performance and characteristics of gas utilities in China. SFA (Aigner et al. 1977; Coelli et al. 2005; Coelli 1996; Meeusen and van den Broeck 1977) is a parametric model that can capture statistical noise and technical inefficiency effects simultaneously. We use Battese and Coelli’s (1995) SFA model specification, because it allows for the incorporation of environmental variables to examine factors influencing technical efficiency. Efficiency is defined as the output of a given firm relative to the output that could be produced by a fully efficient firm using the same input vector. The model applies the general form:

1 $${lnY}_{it}={\beta }_{0}+\beta ln{X}_{it}+({V}_{it}-{U}_{it})$$

where ${Y}_{it}$ is the production (output) of the ${i}^{th}$ decision making unit (DMU) at time *t*, ${X}_{it}$ is a vector of input quantities and $\beta$ is a vector of corresponding parameters to be estimated. ${V}_{it}$ is an error term accounting for statistical noise that cannot be explained by input variables. It is assumed to be independently and identically distributed with mean 0 and variance ${{\sigma }_{v}}^{2}$. ${U}_{it}$ captures the nonnegative technical inefficiency effects in production and is assumed to be independently and identically distributed as truncations at zero of the N (${Z}_{it}\delta$, ${{\sigma }_{u}}^{2}$) distribution. ${Z}_{it}$ is a vector of environmental variables influencing the technical inefficiency, while $\delta$ is a vector of unknown parameters.

The model is estimated using the maximum likelihood method. The parameters in the stochastic production frontier and the technical inefficiency effects are estimated simultaneously. Battese and Coelli (1995) define the level of technical efficiency of production for the ${i}^{th}$ DMU as follows:

2 $${TE}_{it}=\mathrm{exp}\left(-{U}_{it}\right).$$

By definition, ${TE}_{it}$, technical efficiency will take a value between zero and one, and firms with scores closer to 1 are more efficient.

### Data description

The data used in this study is compiled from two data sources: (1) the province-level gas coverage rate and labor in gas distribution sector are collected from the Wind database (https://www.wind.com.cn/), and (2) capital input, gas output, consumer characteristics and macroeconomic status are available from the Information of Development Research Center Website of the State Council (http://www.drcnet.com.cn/). We use a pooled unbalanced panel sample consisting of 24 major province-level divisions in mainland China over the 2006–2015 period, including 179 observations in total. Wind, a financial information service company, collects operation and financial data of 21 major industries in China from the National Bureau of Statistics, the National Development and Reform Commission, the Ministry of Commerce, the General Administration of Customs, and other industry associations. This enables us to collect the province-level data on the gas utilities’ performance as well as their operation process for the period between 2006 and 2015. With ten-year data covering most province-level divisions in mainland China, this paper provides a starting point to evaluate the performance of Chinese gas utilities, incorporating environmental factors to address the reasons for inefficiency in the sector. We use province-level data because it is the only level of recent data that are available.^3^

### Production function model description

Our production function model consists of one output, two types of inputs and four categories of environmental variables. Consider the Cobb Douglas stochastic frontier production function one-step inefficiency effects model specified by Battese and Coelli (1995):

3 $$ln{Y}_{it}={\beta }_{0}+{\beta }_{1}\mathit{ln}\left({K}_{LPG\_it}\right)+{\beta }_{2}\mathit{ln}\left({K}_{natgas\_it}\right)+{\beta }_{3}\mathit{ln}\left({L}_{it}\right)+{V}_{it}-{U}_{it}.$$

where $\beta$ is a vector of unknown parameters to be estimated; $ln{Y}_{it}$ is output that is measured as the natural logarithm (with base e) of total volume of gas (LPG and natural gas) delivered in a year in tons, for the i^th^ province-level division in year t where i = 1,…, I and t = 1, …, T; the inputs are defined as: capital, proxied as length of petroleum pipes in meters (${K}_{LPG\_it})$ and length of natural gas pipes in meters (${K}_{natgas\_it})$; and labor (${L}_{it}$) measured by the total number of staff working in the gas distribution sector. Note that the dataset provides aggregate data of labor without distinguishing labor for LPG distribution and labor for natural gas distribution. Therefore, compared with setting an individual production function for LPG and natural gas respectively, we apply an aggregate production function consisting of one output that is the summation of LPG and natural gas. This is also due to data availability on coverage rate: the dataset includes aggregate data on gas coverage rate only, without distinguishing between natural gas and LPG. The summation of LPG volume and natural gas volume is calculated using the conversion methodology specified by Tu et al. (2007).^4^${V}_{it}$ is an error term reflecting noise and ${U}_{it}$ is a technical inefficiency term with distribution ${N(Z}_{it}\delta ,{{\sigma }_{u}}^{2})$, where ${Z}_{it}$ is a vector of explanatory variables associated with technical inefficiency for utility firms. In addition, ${U}_{it}$ is subtracted since inefficiency results in less output. When ${U}_{it}=0$, the firm is fully efficient and lies on the production frontier. When ${U}_{it}$ is positive, the firm is assumed to be operating below the frontier (Iraizoz et al. 2005).

Literature (Tovar et al. 2015; Hawdon 2003; Goncharuk and lo Storto 2017) has highlighted that some socioeconomic and technological characteristics (i.e., environmental factors) could explain the deviation from the frontier and should be addressed in efficiency analysis. For example, Goncharuk and lo Storto (2017) include the amount of people who benefit from gas supply as one of the determinants of performance as it is one major driver of economies of scale. In this paper, we encompass a similar measure, i.e., coverage ratio (defined as the ratio of the number of urban gas consumers to urban population), in our inefficiency effect specification. In addition, Hollas and Stansell (1988), Li and Phillips (2016), Tovar et al. (2015), and Phillips (2013) incorporate customer density as environmental variable. Rossi (2001) use residential share as environmental variable, and Tovar et al. (2015) show that customer characteristic can affect efficiency level. Following prior literature, we select the factors that may be related to firms’ performance and specify the technical inefficiency effects (the relationship between ${U}_{it}$ and ${Z}_{it}$) as follows:

4 $$\begin{array}{c}{U}_{it}{=\delta }_{0}+{\delta }_{1}\left({GDP}_{it}\right)+{\delta }_{2}\left({coverage}_{it}\right)+{\delta }_{3}\left({hourate}_{LPG\_it}\right)+\\ {\delta }_{4}\left({hourate}_{natgas\_it}\right) + {\delta }_{5}\left({cusden}_{LPG\_it}\right)+{\delta }_{6}\left({cusden}_{natgas\_it}\right)+{W}_{it}\end{array}$$

where δ is an unknown vector of coefficients to be estimated and ${W}_{it}$ is a random variable defined by the truncation of the normal distribution. The environmental factors are: GDP per capita for the i^th^ province-level division in year t (adjusted for inflation based on the year 2006), measured in yuan (${GDP}_{it}$); coverage rate of gas (${coverage}_{it}$), defined as the ratio of the number of urban gas consumers to urban population; household user rate of LPG (${hourate}_{LPG\_it}$) and of natural gas (${hourate}_{natgas\_it}$), defined as the ratio of residential volume to total volume distributed for LPG and for natural gas, respectively^5^; customer density of LPG (${cusden}_{LPG\_it}$) and of natural gas (${cusden}_{natgas\_it}$), measured by the number of customers per length of pipe (persons/m) for LPG and for natural gas. The former two environmental factors reflect the social economic development of different provinces, while the household rate depicts market structure and customer density is associated with the utilization of pipes.

Summary statistics for variables in the stochastic frontier production function are given in Table 1. Note that although the output and the labor input are aggregate variables due to data availability, variables related to market structure, transmission mode and delivery process are distinguished by types of the gas output. For instance, we distinguish between the length of petroleum pipelines and the length of natural gas, because natural gas transmission relies on pipelines, while LPG is transported through both gas cylinders and pipelines.

**Table 1:** Summary statistics

Variable	Mean	Standard deviation	Min	Max
Delivered gas volume	2,366,960.08	2,588,157.60	55,076.14	12,787,098.08
Length of petroleum pipelines	548,813.79	894,212.71	350.00	4,857,000.00
Length of natural gas pipelines	12,215,746.54	10,882,684.61	53,000.00	61,125,370.00
Total labor	7,658.63	5,289.43	706.00	25,200.00
GDP per capita	33,145.39	20,650.89	5,787.00	100,807.04
Coverage rate	0.90	0.10	0.60	1.00
Household user rate of LPG	0.62	0.17	0.14	0.94
Household user rate of natural gas	0.27	0.12	0.05	0.75
Customer density of LPG	91.70	275.41	0.66	2023.43
Customer density of natural gas	0.68	0.27	0.21	2.19

Note that some scholars (Eller et al. 2011) use revenue and cost functions to study revenue efficiency, which is affected by both operational environment and market allocation (Kumbhakar and Lovell 2000). However, government subsidies are widely applied in the Chinese urban gas utilities industry, which may lead to a biased measurement of revenue. For this reason, we apply a production function to study technical efficiency and aim to explore the operational factors that affect utilities’ performance in gas distribution.

Before applying SFA, we need to test for whether or not SFA is an improvement over an Ordinary Least Squares (OLS) regression. A method proposed by Battese and Corra (1977) which defines ${\sigma }^{2}={{\sigma }_{v}}^{2}+{{\sigma }_{u}}^{2}$ and $\gamma ={{\sigma }_{u}}^{2}/{\sigma }^{2}$ from the composite error ${V}_{it}-{U}_{it}$ is used. The model can be used to test whether the technical inefficiency part of the model is necessary. By definition, $\gamma$ ranges from 0 to 1, and a larger value of $\upgamma$ suggests that more of the error term is due to technical inefficiency. Otherwise, OLS would generate consistent estimates. The estimate for $\gamma$ is 0.9999 with a t-ratio of 12142.25, which is statistically significant at the 1% level. This suggests that a large proportion of variation of the composite error term results from inefficiency, so SFA is preferable to OLS in this context.

## Empirical analysis

### SFA Estimation using maximum likelihood

The SF model is estimated using the maximum likelihood that yields a consistent and efficient estimator when the model is well specified. Technical efficiency obtained for the i^th^ province-level division in year t is defined as the general form of Eq. (2). Equations (3) and (4) are estimated using Frontier 4.1 simultaneously, a program written by Coelli (1996).

The results for the production function and inefficiency effects are presented in Table 2. The coefficients for all input variables are significantly positive at the 1% level. For instance, a 1% increase in length of petroleum pipelines results in about 0.13% increase in the gas output whereas a 1% increase in the number of staff leads to about 0.27% increase in the gas output.

**Table 2:** SFA model estimation using maximum likelihood

Variable	Coefficients	t-ratio	Standard error
Inputs			
Ln length of petroleum pipelines **(** ${{\varvec{\beta}}}_{1})$	0.1324***	11.7794	0.0112
Ln length of natural gas pipelines** (** ${{\varvec{\beta}}}_{2}$**)**	0.5025***	13.4052	0.0375
Ln total labor** (** ${{\varvec{\beta}}}_{3}$**)**	0.2728***	4.0360	0.0676
Inefficiency factors			
GDP per capita** (** ${{\varvec{\delta}}}_{1}$**)**	-0.0000***	-4.8794	0.0000
Coverage rate** (** ${{\varvec{\delta}}}_{2}$***)***	0.7398	1.2025	0.6153
Household user rate of LPG **(** ${{\varvec{\delta}}}_{3}$**)**	0.3100*	1.7857	0.1736
Household user rate of natural gas **(** ${{\varvec{\delta}}}_{4}$**)**	1.9667***	6.4114	0.3068
Customer density of LPG **(** ${{\varvec{\delta}}}_{5}$**)**	-0.0008***	-5.3592	0.0001
Customer density of natural gas **(** ${{\varvec{\delta}}}_{6}$**)**	-0.6798***	-5.2762	0.1288

*N* = 179, *T* = 10, cross sections = 24. Unbalanced panel. *, **, and *** denote significance at the 10%, 5%, and 1% level, respectively

As mentioned earlier, given China’s rapid urbanization and industrialization and the challenges involved in meeting its energy supply needs, it is useful to identify environmental factors that influence the performance of gas providers.

The GDP per capita ($GDP$) variable has a negative sign and is significant at the 1% level, implying that prosperous provinces have lower levels of inefficiency (higher levels of efficiency). The result is expected, because the provinces with higher GDP generally have more developed infrastructure and more effective administration. This is important for industries such as water, electricity, gas and telecommunications, which have high levels of requirements for capital investment and governance.

The customer density variables (${cusden}_{LPG}$ and ${cusden}_{natgas}$) have negative coefficients that are statistically significant at the 1% level, suggesting that gas utilities with greater customer density are associated with less inefficiency. Again, this result is expected. Assuming a fixed distribution network, adding more customers should translate into a higher demand for gas. Accordingly, given the fixed input level, the output level of gas should increase. In addition, firms with greater customer density tend to use gas pipelines more sufficiently. Similar results are found in the Chinese urban water sector (Li and Phillips 2016) and in the Chinese electricity sector (Li et al. 2019). This suggests that increasing migration from rural to urban areas may be beneficial to China’s current urban utility system in terms of improving technical efficiency.

Both the household user rate of LPG (${hourate}_{LPG}$) and that of natural gas (${hourate}_{natgas}$) have a positive coefficient. This implies that utilities perform more inefficiently serving residential customers when compared to serving the industry, business, transportation and electric power sectors. This result is consistent with Rossi (2001) who studies the efficiency of the gas distribution sector in Argentina and finds that utilities serving a higher proportion of residential customers are less efficient. An interesting finding is that the extent of the impact of the household user rate on technical inefficiency varies with the output type. The household user rate of natural gas has a larger coefficient and is more statistically significant than that of LPG. This might be related to different transmission modes of gas distribution. Compared with LPG, the network of natural gas delivery requires more costly construction and maintenance to serve residential consumers. Rodríguez Pardina and Rossi (1999) also find that a higher residential delivery ratio increases costs in the natural gas distribution sector. Additionally, unlike other countries, in China, the natural gas price for household is generally lower than the average supply cost and the natural gas price for industry. The residential natural gas consumption in China has been essentially subsidized by the government. Accordingly, firms serving more household customers tend to receive more subsidies from the government and have lower incentives to improve their performance. Table 3 shows the natural gas price comparison for household users and industrial consumers between China and the United States of America from 2011 to 2015. In China, the residential price is much lower than the industrial price, while in the United States of America, the residential price is about double the industrial price.

**Table 3:** Average price of natural gas delivered to residential and industrial consumers for year 2011–2015

	China (unit: yuan/m^3^)		United States (unit: dollars/1000ft^3^)	
	Residential Price	Industrial Price	Residential Price	Industrial Price
2011	2.31	3.27	11.03	5.13
2012	2.36	3.27	10.65	3.88
2013	2.37	3.30	10.32	4.64
2014	2.42	3.52	10.97	5.62
2015	2.42	3.56	10.38	3.93

Source: Natural gas price data is collected from Wind Database and Energy Information Administration (http://tonto.eia.gov/dnav/ng/hist/n3010us3a.htm) respectively

### SFA Estimation using a semi-parametric specification

The estimation of SF models often involves strong parametric assumptions regarding the specification of both the efficient frontier and the inefficiency. There is no evidence about their robustness to the parametric specification of the determinants of inefficiency. Thus, prior literature has relaxed the assumptions and proposed a few non-parametric methods, including Kumbhakar et al. (2007) and Simar et al. (2017) for the efficient frontier and Tran and Tsionas (2009) and Parmeter et al. (2017) for the determinants of inefficiency. However, fully nonparametric estimators may slow rates of convergence.

Accordingly, we attempt to address these issues by adopting a semi-parametric model proposed by Forchini and Theler (2023). This method relaxes the parametric assumptions in specifying the inefficiency of a stochastic frontier model and imposes weaker assumptions on the inefficiency. In addition, without incurring the curse of dimensionality as a fully non-parametric approach, it leads to a fast and accurate estimator of the inefficiency.

The results of this semi-parametric approach are presented in Table 4. The coefficients for all input variables are significantly positive at the 1% level. In addition, most estimated coefficients of inefficiency factors are consistent with the main result (Table 2), while coverage rate of gas (${coverage}_{it}$) is significantly positive. This implies that the provinces with a large ratio of urban gas consumers to urban population have higher levels of inefficiency (lower levels of efficiency). This result may reflect the operation inefficiency as the serving consumers increases.

**Table 4:** SFA model estimation using a semi-parametric specification

Variable	Coefficients	t-ratio	Standard error
Inputs			
Ln length of petroleum pipelines **(** ${{\varvec{\beta}}}_{1})$	0.1581***	14.95726	0.0106
Ln length of natural gas pipelines** (** ${{\varvec{\beta}}}_{2}$**)**	0.5252***	16.48089	0.0319
Ln total labor** (** ${{\varvec{\beta}}}_{3}$**)**	0.3815***	27.29706	0.014
Inefficiency factors			
GDP per capita** (** ${{\varvec{\delta}}}_{1}$**)**	-0.001***	-4.71564	0.0002
Coverage rate** (** ${{\varvec{\delta}}}_{2}$***)***	0.0694***	13.21137	0.0053
Household user rate of LPG **(** ${{\varvec{\delta}}}_{3}$**)**	0.7416***	241.9475	0.0031
Household user rate of natural gas **(** ${{\varvec{\delta}}}_{4}$**)**	0.6482***	68.57769	0.0095
Customer density of LPG **(** ${{\varvec{\delta}}}_{5}$**)**	-0.0956***	-3.93828	0.0243
Customer density of natural gas **(** ${{\varvec{\delta}}}_{6}$**)**	-0.126***	-44.9612	0.0028

### OLS Estimation

To examine the sensitivity of our production function, we also compare our results with that of an alternative specification without environmental factors obtained from ordinary least squares estimation. The results without environmental variables are listed in Table 5. As expected, all inputs are significant and positively correlated with output, which are consistent with our main results from SFA estimation.

**Table 5:** Ordinary least squares estimation results of production function

	Coefficients	t-ratio	Standard error
Ln length of petroleum pipelines	0.0915***	3.8626	0.0237
Ln length of natural gas pipelines	0.5914***	14.0264	0.0422
Ln total labor	0.4092***	5.9269	0.0690

*N* = 179, *T* = 10 (from 2006 to 2015), cross sections = 24 (province-level divisions). Unbalanced panel. *, **, and *** denote significance at the 10%, 5%, and 1% level, respectively

### Discussion

**Fig. 1:**
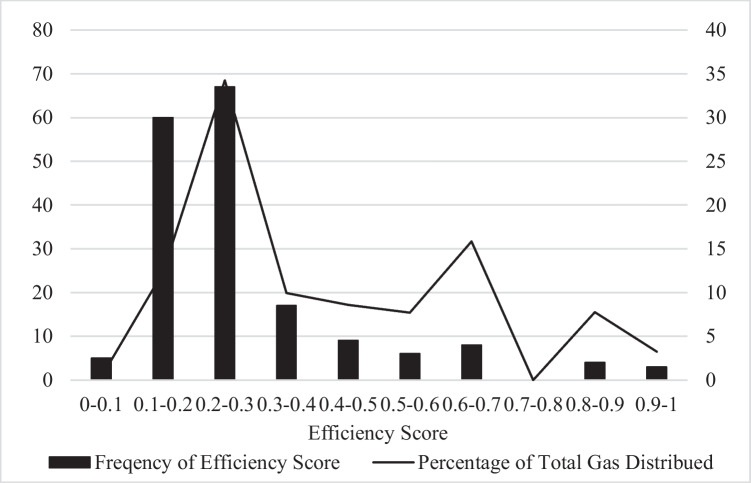
The frequency distribution of technical efficiency scores. Note: The left-hand side axis shows the distribution of technical efficiency scores (%). The right-hand side axis shows the percentage of gas delivered for gas providers with corresponding efficiency scores (%)

The frequency distribution of technical efficiency scores and the percentage of total gas delivered can be seen in Fig. 1. Most samples cluster far below the frontier with technical efficiency scores from 0.1 to 0.4. These relatively inefficient provinces deliver most of the gas in China although a small peak of gas distribution occurs at provinces with efficiency scores around 0.65. This indicates that improving efficiency of gas distribution can provide an important avenue for China to meet its goal of increasing energy supply.

**Fig. 2:**
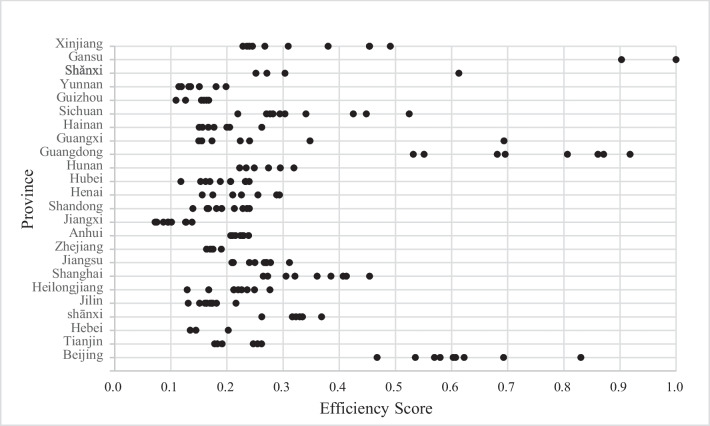
Technical efficiency scores of each province over the 2006–2015 period

Technical efficiency scores for 24 province-level divisions are plotted in Fig. 2. The average technical efficiency score of all samples is 0.2954. The score of each province over the 2006–2015 period ranges from 0.0721 to 0.9997. This means that the most inefficient province could reduce usage of inputs by 92.79%. Moreover, Table 6 presents the technical efficiency scores for 24 province-level divisions over time. As can be seen, year 2006–2015 have seen an improvement in technical efficiency overall, except a few provinces. This can be explained by China’s rapid urbanization and economic development over time. In addition, there is no clear pattern between technical efficiency and the endowment of oil and natural gas. On one hand, Beijing, Gansu, Shanxi, Sichuan and Xinjiang take advantage of their large endowment of oil and natural gas to distribute gas with high efficiency. On the other hand, Heilongjiang, Tianjin, Jilin and Shandong are less efficient, although they have a large amount of oil and natural gas deposits. Compared with the endowment, the local socio-economic status is more related to a high level of technical efficiency, which is also indicated by the estimated result of the GDP per capita ($GDP$) variable (Table 2).

**Table 6:** Technical efficiency across provinces over time

	2006	2007	2008	2009	2010	2011	2012	2013	2014	2015
Xinjiang	0.2402	0.2286	0.2359	0.2457	0.2678	0.3093	0.3803	0.4912	0.4539	0.4542
Gansu				0.9028	0.9997					
Shǎnxi	0.3035	0.2517	0.2713							0.6131
Yunnan				0.1806	0.1985	0.1324	0.1355	0.151	0.1193	0.1145
Guizhou	0.1638	0.1678	0.1589	0.1546	0.1265	0.1094				
Sichuan	0.4482	0.5247	0.4251	0.3406	0.2943	0.3034	0.2707	0.2771	0.2822	0.2195
Hainan				0.1669	0.1563	0.1772	0.1506		0.1998	0.2622
Guangxi		0.3482	0.2238	0.1736		0.1499	0.1556	0.2050	0.2404	
Guangdong	0.8608	0.8710		0.532	0.5513	0.9179	0.8065	0.6937	0.6957	0.6815
Hunan			0.2226	0.2345	0.2488	0.2741	0.295		0.3196	
Hubei	0.118	0.1699	0.1623	0.1533	0.1885	0.2064	0.2068	0.2399	0.2328	0.2339
Henai			0.1563	0.175	0.2104	0.226	0.2555	0.2886	0.2936	
Shangdong	0.1646	0.1393	0.1908	0.1673	0.1814	0.2134	0.2284	0.2401		0.2359
Jiangxi		0.1015	0.0948	0.0721	0.0755	0.0869	0.0958	0.1264	0.1381	0.1276
Anhui			0.2241	0.2069	0.2098	0.2085	0.2153	0.2285	0.2387	0.2298
Zhejiang				0.1637	0.1642	0.1705	0.1717	0.1726	0.1901	0.1754
Jiangsu	0.2102	0.24	0.2091	0.2115	0.2500	0.2707	0.2699	0.2664	0.2779	0.3115
Shanghai		0.3055	0.2727	0.2647	0.3213	0.3608	0.3852	0.4069	0.4129	0.4542
Heilongjiang	0.2765		0.1292	0.1676	0.2123	0.2203	0.2128	0.2490	0.2360	0.2263
Jilin	0.1310	0.1515	0.1700	0.1633	0.1636	0.1606	0.1724	0.1816	0.2161	0.1745
Shānxi	0.2623		0.3298	0.3167	0.3232	0.3685	0.3345			
Hebei				0.1448	0.1349	0.2026				
Tianjin				0.1783	0.1914	0.1834	0.2469	0.2546	0.2618	
Beijing	0.4677		0.6030	0.5695	0.5800	0.5355	0.6074	0.6226	0.6929	0.8302

Moreover, a heterogeneity analysis is applied to explore the impact of geographical region on technical efficiency. Specifically, we included a region dummy (North) and the interaction terms between the inputs and the region dummy (North) to the model. The results (Table 7) show that the coefficient of the interaction term, $$Ln length of natural gas pipelines \times North ({\beta }_{6})$$, is significantly positive while the coefficient of $$Ln\;length\;of\;petroleum\;pipelines\;\times North ({\beta }_{5})$$ is insignificant, suggesting that increasing one unit of natural gas pipe in Northern China yields higher output than in Southern China. The results are expected because the northern areas are centrally heated in winter, and natural gas (as well as coal) is the main energy source for central heating. The result is consistent with the energy demand in China. However, the coefficient of $$Ln total labor \times North ({\beta }_{7})$$ is significantly negative, indicating that identical amount of labor supply delivers more output in Southern China than Northern China, probably because cities with high economic competitiveness are mostly concentrated in the South and thus employees are expected to achieve higher productivity. The estimates of environmental variables remain consistent overall.

**Table 7:** Heterogeneity analysis

Variable	Coefficients	t-ratio	Standard error
Inputs			
Ln length of petroleum pipelines (${{\varvec{\beta}}}_{1})$	0.1031***	3.0669	0.0336
Ln length of natural gas pipelines (${{\varvec{\beta}}}_{2}$)	0.5302***	9.6788	0.0548
Ln total labor (${{\varvec{\beta}}}_{3}$)	0.4250***	4.8317	0.0879
North (${{\varvec{\beta}}}_{4}$)	0.1130	0.1129	1.0008
Ln length of petroleum pipelines × North (${{\varvec{\beta}}}_{5})$	-0.0094	-0.2498	0.0374
Ln length of natural gas pipelines × North (${{\varvec{\beta}}}_{6}$)	0.2199***	2.0799	0.1057
Ln total labor × North (${{\varvec{\beta}}}_{7}$)	-0.4156***	-2.9325	0.1417
Inefficiency factors			
GDP per capita (${{\varvec{\delta}}}_{1}$)	0.0000***	-3.4908	0.0000
Coverage rate (${{\varvec{\delta}}}_{2}$)	0.8282	1.5846	0.5227
Household user rate of LPG (${{\varvec{\delta}}}_{3}$)	0.2829	1.2947	0.2185
Household user rate of natural gas (${{\varvec{\delta}}}_{4}$)	1.4283***	4.3321	0.3297
Customer density of LPG (${{\varvec{\delta}}}_{5}$)	-0.0005***	-2.3882	0.0002
Customer density of natural gas (${{\varvec{\delta}}}_{6}$)	-0.6610***	-3.6930	0.1790

## Concluding observations and policy implications

This paper examines inefficiency in the Chinese gas utilities industry. According to our results, there is a large range of efficiency across Chinese firms, which implies that improving efficiency can provide a crucial avenue for China to increase its energy supply. GDP per capita and high levels of customer density are associated with higher levels of efficiency, while utility firms serving larger proportions of household customers are associated with lower levels of efficiency. These are all variables representing factors that are beyond the control of a manager, but that can be affected by government decisions related to city planning and the allocation of energy resources. In contrast, we find no clear pattern between technical efficiency and the endowment of oil and natural gas, which indirectly indicates that the technical efficiency of the Chinese gas utilities can be improved by more appropriate regulation and policy change. Indeed, gas utilities in provinces with higher GDP per capita deliver significantly better performance. An important policy implication of this paper is that gas distribution can be performed more efficiently in areas with higher customer density, which are expected to increase as China becomes more urbanized. Additionally, our results suggest that the pricing structure of the residential sector compared to other sectors may be incentivizing inefficient use of natural gas and petroleum.

The world has witnessed China's rapid economic development in the past decades and its continued growth in recent years. However, the rapid urbanization of China has presented several challenges. Providing heat to large cities using coal has negative implications for air quality and living standards. As clean energy supply, LPG and natural gas can help alleviate these problems. In addition, the increasing risks in the scarcity in gas supply could hinder China’s sustainable development. Thus, it is crucial to introduce incentive-based performance regulation to the gas sector, which has been implemented in many sectors, such as water, electricity and telecommunication, to improve the performance of local monopolies. As shown by this study, there are several gas utilities in China that could benefit from benchmarking. Identifying the best and worst performers is the key to improving the country’s supply of energy. In summary, the findings of the paper can point out where gas use is efficient and which factors can drive the improvement of technical efficiency. This information combined with an ETS program should help China figure out how to deal with the competing goals of having rapid urbanization and trying to achieve their Paris Agreement pledge.

This study has several limitations. Firstly, due to lack of data, this study applies province-level data. Future studies should use utility-level data to study the efficiency of natural gas and the liquefied petroleum gas sector when more microscopic data become available. Furthermore, the study should consider additional factors at the utility level that cause inefficiencies, including ownership, market share, management skills, and employee structure. These factors have been extensively addressed in the existing literature on efficiency (Ertürk and Türüt-Aşık 2011; Tovar et al. 2015; Li and Phillips 2016; Li et al. 2019). For instance, research by Eller et al. (2011) reveals that national oil companies exhibit lower efficiency compared to private international oil companies, indicating the adverse impact of government ownership on efficiency. Additionally, besides focusing on technology efficiency, it is crucial to explore the role of scale economics, an important aspect often examined in previous studies on environmental and resource economics (e.g., Erbetta and Rappuoli 2008; Goncharuk and lo Storto 2017). Therefore, future studies are encouraged to investigate scale economics within China’s gas sector.

## Data Availability

The datasets used in this study are available from the Wind database (https://www.wind.com.cn/) and the Information of Development Research Center Website of the State Council (http://www.drcnet.com.cn/).

## Footnotes

1 An autonomous region is the highest level of a minority autonomous entity in China. It has a comparably higher population of a designated minority ethnic group and a relatively lower level of economic development. A direct-controlled municipality is under the direct administration of the central government, which has the same rank as a province.

2 In China, first-level administrative divisions include provinces, autonomous regions, direct-controlled municipalities and special administrative regions.

3 Although Hu (2014) used the utility-level data to evaluate energy efficiency of China’s energy sector, the data period is year 2000–2005.

4 Tu et al. (2007) collected samples from the distribution stations of China’s National Petroleum Corporation and estimated the average of molar density of natural gas, which can be used to convert the unit of natural gas to ton.

5 The users of gas consist of the residential, industrial, commercial, transportation and electric power sectors.
